# MiR-520a-3p Inhibited Macrophage Polarization and Promoted the Development of Atherosclerosis via Targeting UVRAG in Apolipoprotein E Knockout Mice

**DOI:** 10.3389/fmolb.2020.621324

**Published:** 2021-03-09

**Authors:** Jing Rui Qi, Dian Ru Zhao, Li Zhao, Fan Luo, Mei Yang

**Affiliations:** ^1^ Department of Geratology, Cangzhou Central Hospital, Cangzhou, China; ^2^ Xinxiang Medical University, Xinxiang, China

**Keywords:** MiR-520a-3p, macrophage polarization, autophagy, atherosclerosis, chronic inflammatory blood vessel disease

## Abstract

Atherosclerosis (AS), a kind of chronic inflammatory blood vessel disease, is a main cause of cardiovascular disease, which is a leading cause of mortality around the world. Accumulation of macrophages induced by inflammation contributes to AS development. It has been indicated that microRNAs (miRNAs) are involved in the process of AS. However, the pathway and gene miRNAs targeting are poorly understood. Here we reported that miR-520a-3p was increased in mice with AS and silencing of miR-520a-3p attenuated AS process. Furthermore, inhibition of miR-520a-3p increased the expression of α-SMA and collagen. In addition, miR-520a-3p silencing inhibited the expression of M1 macrophage polarization markers and pro-inflammatory genes and promoted the M2 macrophage polarization. What’s more, forced expression of miR-520a-3p diminished IL4/IL13 induced macrophage autophagy via targeting UVRAG. Collectively, our study reveals the role of miR-520a-3p in macrophage polarization and suggests the potential of miRNA as a novel treatment target of AS.

## Introduction

Atherosclerosis, as a chronic progressive disease, develops mainly in large or medium-sized arteries, such as aorta, coronary artery, cerebral artery and lower extremity artery (Fatkhullina et al., 2016). Acute myocardial infraction, stroke (cerebral infarction and hemorrhage) and peripheral arterial diseases caused by atherosclerosis development and vulnerable plaque rupture are the leading cause of human death and disability (Mukherjee, 2020). Nowadays, it is well believed that multiple factors are involved in atherosclerosis formation and development, including abnormal lipid metabolism, inflammation, immune disorder (Fioranelli et al., 2018). Recent evidence also proves that epigenetic processes can take part in the atherosclerosis progression, such as DNA methylation abnormalities, histone modification and noncoding RNAs (Xu et al., 2018).

There are many cells which contribute to the development of atherosclerosis, especially macrophage (Koelwyn et al., 2018). Macrophage infiltration in the lesion is the hallmark of the atherosclerosis (Tabas and Bornfeldt, 2016). Macrophages transform to foam cells by uptake excess lipid and following apoptosis which contributes to the necrotic core formation and plaque instability (Tabas and signaling, 2009). As typical immune cells, macrophage associated inflammation also aggravates the plaque burden (Colin et al., 2014).

However, recent studies have proved the heterogeneity and plasticity of macrophage in atherosclerosis. According to respond to the different micro-environmental signals such as cytokines, macrophage can differentiate into pro-inflammatory M1 subsets and anti-inflammatory M2 subsets (Yang et al., 2020). M1 macrophages activated mainly by Th-1 cytokines are responsible for the sustained inflammatory cytokines release as well as some other toxic mediators such as NO, ROS and MMP, which together contributes to the plaque progression (Rath et al., 2014). Alternatively, activated M2 macrophages mainly by IL-4 and IL-13 are a group of anti-inflammatory cells which counteract to the M1 macrophage function. M2 macrophages are actively involved in IL-10 production, tissue repair, phagocytosis and collagen production which improve the plaque status. Accordingly, balance between the M1 phenotype and M2 phenotype in plaque is a critical guide to treat atherosclerosis (Chinetti-Gbaguidi et al., 2015).

• MicroRNAs (miRNAs), a kind of small single stranded RNA about 18–24 nucleotide length, are highly conserved in evolution in animals, plants and virus(Bartel, 2009). MiRNAs function by mediating the degradation of target gene through binding with 3’-UTR of target genes (Bartel, 2004). In the past 15 years, more and more studies investigated the roles of miRNAs in development, regeneration, inflammation, immunity and metabolism related disease(Singh et al., 2013; Bhaskaran and Mohan, 2014; Di Leva et al., 2014; Sen and Ghatak, 2015; Vienberg et al., 2017). Previous studies showed the role of miR-520a-3p in cancer development and neurodegeneration, but the function of miR-520a-3p in heart disease has not been well described(Surgucheva et al., 2013; Li et al., 2017; Zhang et al., 2017).
• Here we reported that miR-520a-3p was increased in mice with AS and silencing of miR-520a-3p attenuated AS process. Furthermore, inhibition of miR-520a-3p increased the expression of collagen and α-SMA. In addition, miR-520a-3p silencing inhibited the expression of M1 macrophage polarization markers and pro-inflammatory genes and promoted the M2 macrophage polarization. What’s more, forced expression of miR-520a-3p diminished IL4/IL13 induced macrophage autophagy via targeting UVRAG.

## Methods

### Establishment of Mice Atherosclerosis Model

All the animals are obtaining from Beijing Vital River Laboratory Animal Technology Co., Ltd., (Beijing, China) and bred in SPF animal housing room. All animal experimental procedures are performed according to the protocols of Cangzhou Central Hospital Animal Care and Use Committee and conformed to the “Guide for the Care and Use of Laboratory Animals” of the National Institute of Health in China. Male C57BL/6 apoE^-/-^ mice are housed until 6-8 weeks old randomly separated into four group: sham group (feeding with normal diet), and atherosclerosis group (AS, feeding with a western type diet (34.16% sugar, 21% fat, 19.5% casein and 0.25% cholesterol) for 3 months to develop atherosclerosis model), AS+antagomir-520a-3p group and AS + antagomir-NC group. To explore the role of miR-520a-3p in atherosclerosis process in mice, we inject adenovirus to increase the expression level of miR-520a-3p mimics and negative control (Genepharma Co. Shanghai, China) via tail vein injection to the mice twice a week for the last 4 weeks of a 12 week western type diet (WD) feeding program.

### Atherosclerosis Analysis

After 12 weeks of WD diet, mice are anesthetized using pentobarbital sodium via intraperitoneal injection. 20  ml of phos-phate-buffered saline (PBS) are used to perfuse hearts. Then, total aorta is dissected and fixed with 10% buffered formalin for 24  h. We use paraffin to embed total aorta and cut as 6 μM section. We use 0.3% oil red-O to stain lipids for 2  h and 78% methanol is used to destain for 5  min. H and E staining was performed to evaluate the plaque area. Plaques are analyzed by Olympus microscope. We used Image-J software to quantify the positive staining area and evaluate lipid accumulation level and plaque area. Lipid and plaque area of each mouse are quantified as the average of 5 sections of positive staining areas from one animal.

### Cell Culture and Transfection

Peritoneal macrophages were harvested under aseptic conditions at 3 days after i.p., injection with 3% thioglycollate (Sigma-Aldrich). Red blood cells were lysed in erythrocyte lysis buffer (10 mmol/L Tris-HCl, pH 7.2), containing 150 mmol/L NH_4_Cl. The resultant peritoneal macrophages were seeded in 6-well tissue culture plates at 1 × 106 cells/well in 1640 medium containing 10% FBS and 4 hours later the nonadherent cells were removed. The macrophages were then cultured for 24 h in DMEM medium containing 1% FBS before treatment with IL4 (10 ng/ml) and IL13 (10 ng/ml) in serum free DMEM. UVRAG protein factor (5 ng/ml) was administrated together with IL4/IL13. After 24 h treatment, the macrophages were collected for the further analysis. MiRNA-520a-3p mimics and inhibitor (AMO-520a-3p) (Ribobio, Guangzhou) are transfected into macrophages by lipofectamine 3,000 and cultured for 6 h before the treatment of IL4/IL13.

### qRT-PCR

TRIzol reagent (Invitrogen) is used to extract total RNA from cells and tissues according to the manufacture protocol. Concentrations of total RNA were quantified by Nanodrop 2,000 software (Nanodrop Products) and 500 ng total RNA was converted to cDNA by the iScript cDNA Synthesis Kit (Bio-Rad). qPCR was performed using SYBR Green (Thermo Fisher Scientific). The reaction was performed on ABI7300 Real-time PCR system. Melting curve analysis was used to guarantee the specificity of primers. The mRNA levels were normalized to GAPDH as an internal control. ΔΔCt method was used to indicate the relative expression level of corresponding genes.

### Western Blot

Protein samples are isolated from aorta tissue and macrophages using RIPA buffer and normalized to same concentration by PBS and loading buffer. Samples are boiled at 100°C for 10 mins and separated on 12% SDS-PAGE gels. Nitrocellulose (NC) membranes are used for transmembrane. 5% non-fat milk is used for NC membranes blocking. First antibody is incubated over night at 4°C. The second antibody is incubated at room temperature for 1 h. NC membranes are scanned using Odyssey System. Image Studio software was used to analyze the results that normalized with respect to loading control.

The antibodies are listed as follows:

Anti-α-SMA antibody (ab5694, 1:1000) and anti-collagen I antibody (ab34710, 1:1000) are obtained from abcam (Cambridge, United Kingdom). Anti-LC3 antibody (L8918, 1:2000) is obtained from Sigma-Aldrich (Darmstadt, Germany). Anti-CD206 antibody (91992, 1:1000), anti-Arg1 antibody (93668, 1:1000) and anti-p62 antibody (23214, 1:1000) are obtained from Cell Signaling Technology (Danvers, MA, United States). The secondary antibodies IRDye700/800 Rabbit or Mouse were obtained from LICOR (Lincoln, Nebraska, United States).

### Immunofluorescence Staining

Frozen sections are washed by PBS for 3 times and fixed using 4% paraformaldehyde (Beyotime, Beijing, China) at room temperature for 15 mins. 0.4% Triton X-100 is used for penetration and 5% Goat serum is used to block sample sections. First antibody is incubated at 4°C. The second antibody is incubated at room temperature for 1 h.

### Masson Staining

Paraffin sections are prepared for masson staining and total experiment is performed using masson staining kit (Solarbio, Beijing, China) and followed the protocol provide by manufacturer.

### Luciferase Assay

To detect the interaction between miR-520a-3p and UBRAG, wildtype UVRAG-3’UTR or mutant UVRAG -3’UTR sequences that contain the putative binding sites of miR-520a-3p is inserted into psiCHECK-2 luciferase reporter plasmid. HEK293 cells are transfected with miR-CTL or miR-520a-3p mimics with reporter vectors. Luciferase activity are detected after 48 h post-transfection (Promega).

### Statistical Analysis

Statistical analyses were assessed with t-test or one-way ANOVA. All the data are presented as means ± SEM. We used GraphPad Prism 8.0 for all statistics.

## Results

Silencing of miR-520a-3p inhibited atherosclerosis process.

**FIGURE 1:**
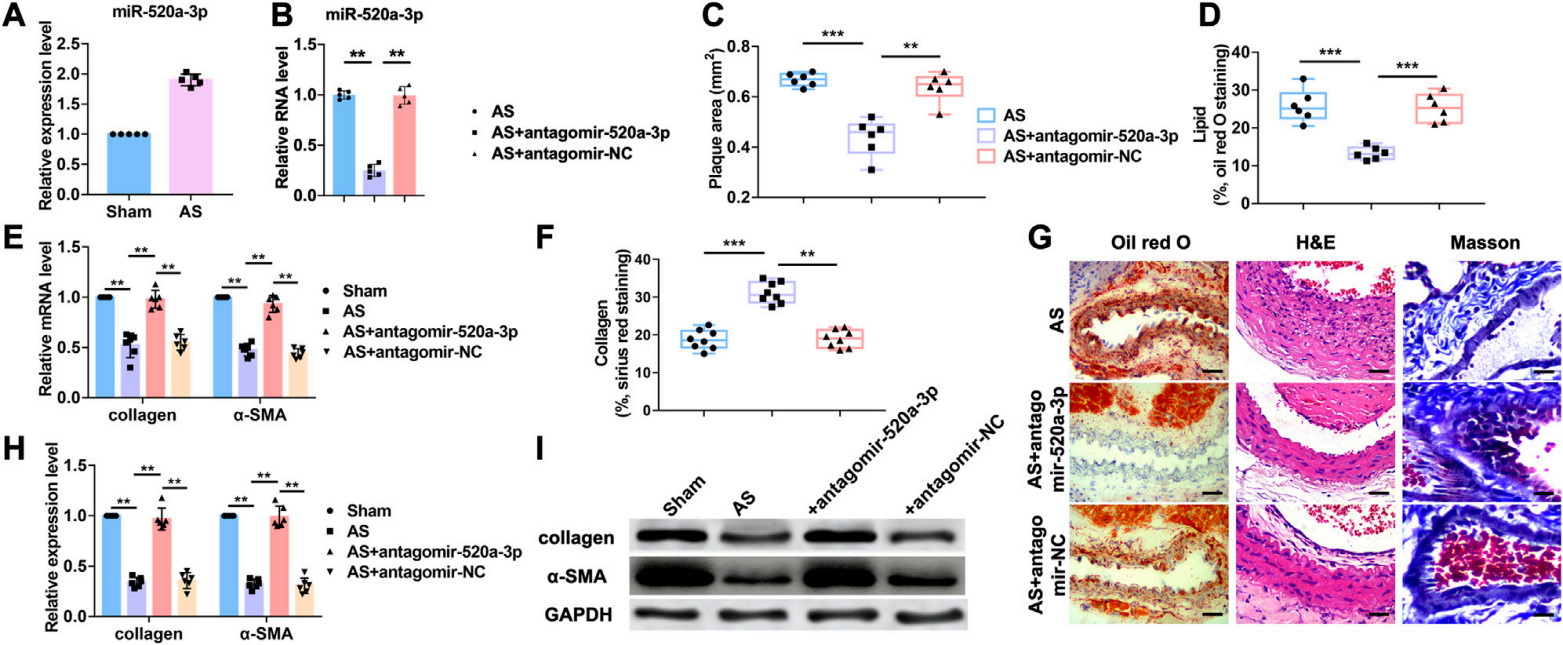
Inhibition of miR-520a-3p attenuated the development of atherosclerosis. **(A)** RT-PCR analysis showing increased level of miR-520a-3p in plaque and artery tissue in apoE-/- mice. Data are mean ± SEM; Two-tailed t test was used for the statistical analysis. n=5 mice per group; **(B)** RT-PCR analysis showing the expression level of miR-520a-3p after treatment of antagomir-520a-3p in plaque and artery tissue in apoE-/- mice. Data are mean ± SEM; one-way ANOVA was used for the statistical analysis. n=5 mice per group; **(C)** Plaque area was evaluated by H and E staining. Data are mean ± SEM; one-way ANOVA was used for the statistical analysis. n=6 mice per group; **(D)** The level of accumulated lipid was measured via oil red O staining. Data are mean ± SEM; one-way ANOVA was used for the statistical analysis. n=6 mice per group; **(E)** RT-PCR analysis showed increased collagen and α-SMA after miR-520a-3p inhibition in plaque and artery tissue in apoE-/- mice. Data are mean ± SEM; one-way ANOVA was used for the statistical analysis. n=6 mice per group; **(F)** Collagen deposition was examined by Sirius red staining. Data are mean ± SEM; one-way ANOVA was used for the statistical analysis. n=8 ice per group; **(G)** images of oil red O, H and E and Masson staining. The scar bar represents 50 μm; **(H)** and; **(I)** Western blot analysis showed increased collagen and α-SMA after miR-520a-3p inhibition in plaque and artery tissue in apoE-/- mice. Data are mean ± SEM; one-way ANOVA was used for the statistical analysis. n=6 mice per group. **P<0.01, ***P<0.001.

The functions of microRNAs (miRNAs) are well studied over the last 15 years. However, the roles of miRNAs in atherosclerosis are poorly understood. Our previous study indicated that miR-520a-3p was elevated in apoE^-/-^ mice with atherosclerosis (Figure 1A). To further investigate the role of miR-520a-3p in atherosclerosis, atherosclerosis mice model was established as described in apoE^-/-^ mice. Antagomir-520a-3p was administrated via tail vein injection to knockdown the expression level of miR-520a-3p (Figure 1B). First, HE stainning was performed to measure the plaque area (Figure 1C G). As shown in Figure 1C G, silencing of miR-520a-3p by specific antagomiRNA reduced the plaque area of lesions in apoE^-/-^ mice with atherosclerosis. Furthermore, histologic analysis showed that inhibition of miR-520a-3p expression significantly inhibited lipid deposition and elevated collagen level (Figure 1D F G). RT-PCR analysis detected the mRNA levels of collagen I and α-SMA and we found that silencing of miR-520a-3p showed significant increase of mRNA levels of collagen and α-SMA in lesions in apoE^-/-^ mice with atherosclerosis (Figure 1E). In addition, collagen and α-SMA protein levels were also examined through western blot. As shown in Figure 1H I, miR-520a-3p inhibition elevated the expression levels of α-SMA and collagen, which were inhibited in lesions in apoE^-/-^ mice with atherosclerosis. These results indicated that silencing of miR-520a-3p attenuated atherosclerosis progression.

### Silencing of miR-520a-3p Inhibited Inflammation Effect

**FIGURE 2:**
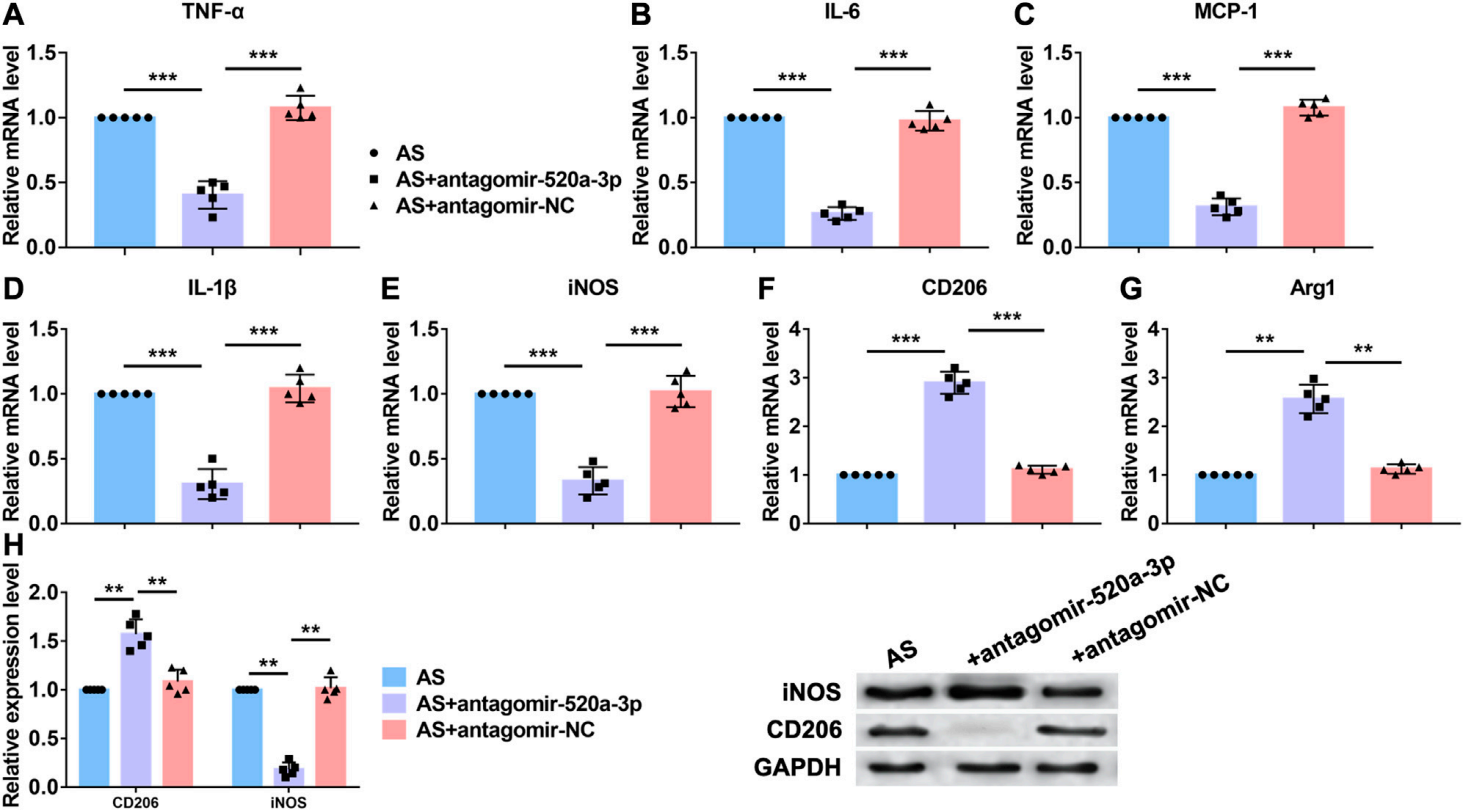
MiR-520a-3p silencing inhibited macrophage polarization to M1 subset. **(A)** RT-PCR analysis showed the mRNA levels of macrophage polarization related markers TNF-α; **(B)** IL-6; **(C)** MCP-1; **(D)** IL-1β; **(E)** INOS; **(F)** CD206; and **(G)** Arg1 in plaque and artery tissue in apoE-/- mice. Data are mean ± SEM; one-way ANOVA was used for the statistical analysis. n=5 mice per group; **(H)** Western blot analysis showed increased CD206 and decreased INOS after miR-520a-3p inhibition in plaque and artery tissue in apoE-/- mice. Data are mean ± SEM; one-way ANOVA was used for the statistical analysis. n=5 mice per group. **P<0.01, ***P<0.001.

Acting as a critical inflammation regulatory cell, macrophages contribute to atherosclerosis progression. Lipid recognition and uptake of macrophages lead to the formation of foam cells. In response to multiple microenvironment signals, the subsets of activated macrophages exhibit distinct pathologic roles in atherogenesis. Here we investigated whether miR-520a-3p was involved in the inflammation response that regulated by macrophages. First, RT-PCR analysis was performed to examine the mRNA levels of inflammatory cytokines that were increased in atherosclerosis progression. As shown in Figure 2A–E, silencing of miR-520a-3p inhibited the mRNA levels of TNF-α, IL-6, MCP-1, IL-1β and iNOS and suggested the inhibition of proinflammatory(M1) macrophage polarization. Anti-inflammatory(M2) macrophage is another subset of macrophages. The activation of M2 macrophages was also detected using RT-PCR. As shown in Figure 2F G, two M2 macrophage markers, CD206 and Arg1, were elevated after inhibition of miR-520a-3p expression. Besides, protein levels of CD206 and iNOS were also measured. Silencing of miR-520a-3p showed significant inhibitory effect on iNOS expression and increased CD206 level (Figure 2H). These data suggested that miR-520a-3p contributed to the suppression of M2 macrophage polarization and activation of M1 macrophages.

MiR-520a-3p inhibited Macrophage polarization by inhibiting autophagy level.

**FIGURE 3:**
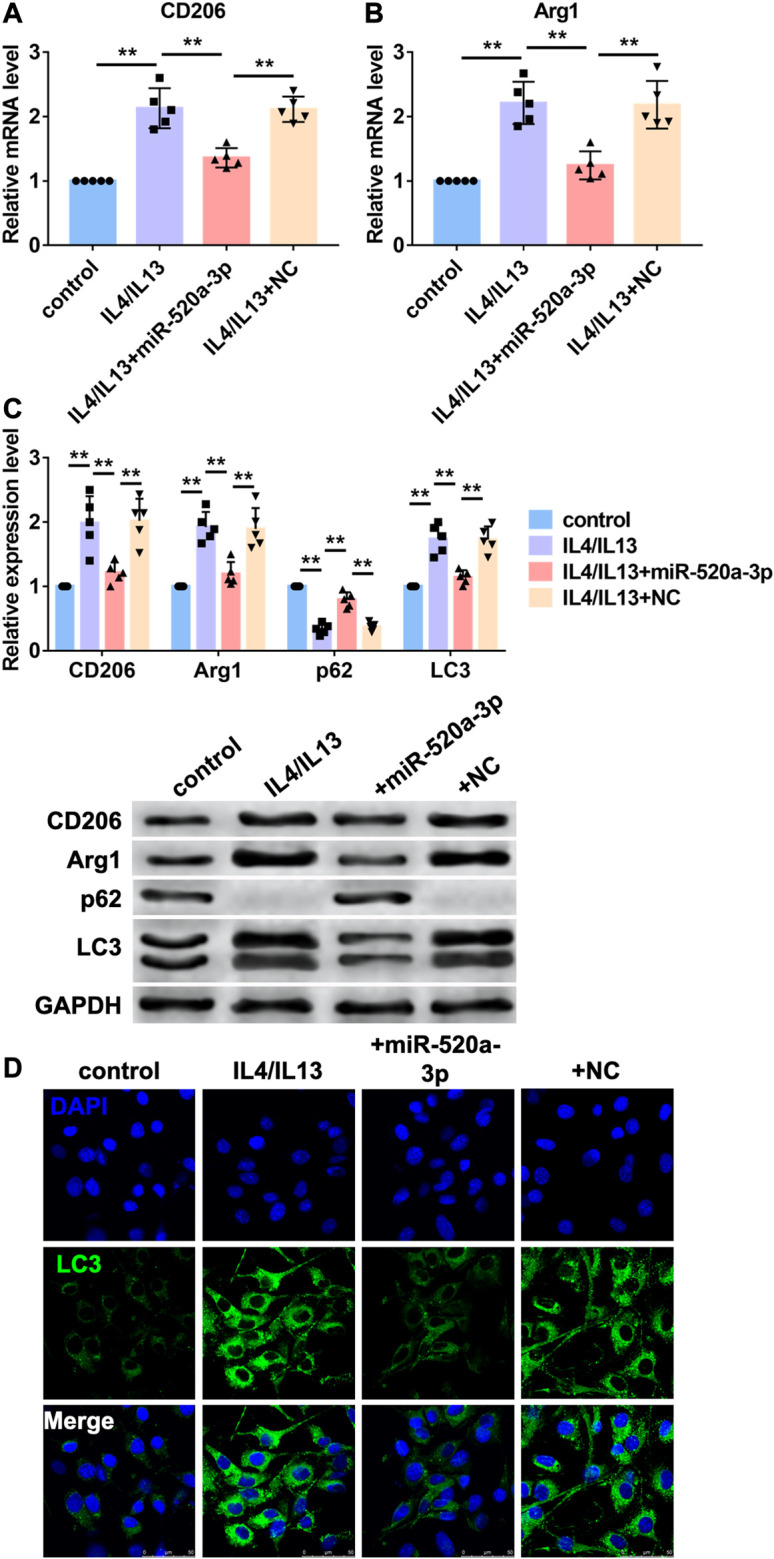
MiR-520a-3p inhibited macrophage autophagy and M2 subset polarization. **(A)** and; **(B)** RT-PCR analysis showed the mRNA levels of CD206 and Arg1 in macrophages. Data are mean ± SEM; one-way ANOVA was used for the statistical analysis. n=5 independent cell cultures; **(C)** Western blot analysis showed the protein levels of CD206 and Arg1 in macrophages. Data are mean ± SEM; one-way ANOVA was used for the statistical analysis. n=5 independent cell cultures; **(D)** Immunofluorescence images showed the LC3 level in macrophages. Scale bars represent 50 μm. **P<0.01.

Then, we investigated the mechanism of miR-520a-3p regulating macrophage polarization in vitro. Bone marrow derived macrophages (BMDMs) were treated with IL4/IL13 to induce M2 macrophages polarization. MiR-520a-3p mimics were transfected into BMDMs to overexpress miR-520a-3p. First, RT-PCR was used to measure mRNA levels of two markers of M2 macrophages polarization, CD206 and Arg1. As shown in Figure 3A B, treatment of IL4/IL13 significantly increased mRNA level s of CD206 and Arg1 and overexpression of miR-520a-3p reduced the mRNA level s of CD206 and Arg1. In addition, western blot analysis also showed that miR-520a-3p forced expression inhibited IL4/IL13 triggered increase of protein levels of CD206 and Arg1 (Figure 3C). Previous study indicated that autophagy contributed to M2 macrophages polarization. Here we detected the autophagy related degradation substrate, p62, and the classic marker of autophagy, LC3. As shown in Figure 3C, treatment of IL4/IL13 increased LC3 level and promoted the degradation of p62. Overexpression of miR-520a-3p reversed that effect and inhibited the expression of LC3 and degradation of p62 (Figure 3C). Then, immunostaining was performed to measure the change of autophagy by LC3 staining. IL4/IL13 treatment promoted autophagy process and forced expression of miR-520a-3p inhibited that process (Figure 3D). These data suggested that miR-520a-3p inhibited M2 macrophage polarization by inhibiting cell autophagy.

UVRAG is a target of miR-520a-3p.

**FIGURE 4:**
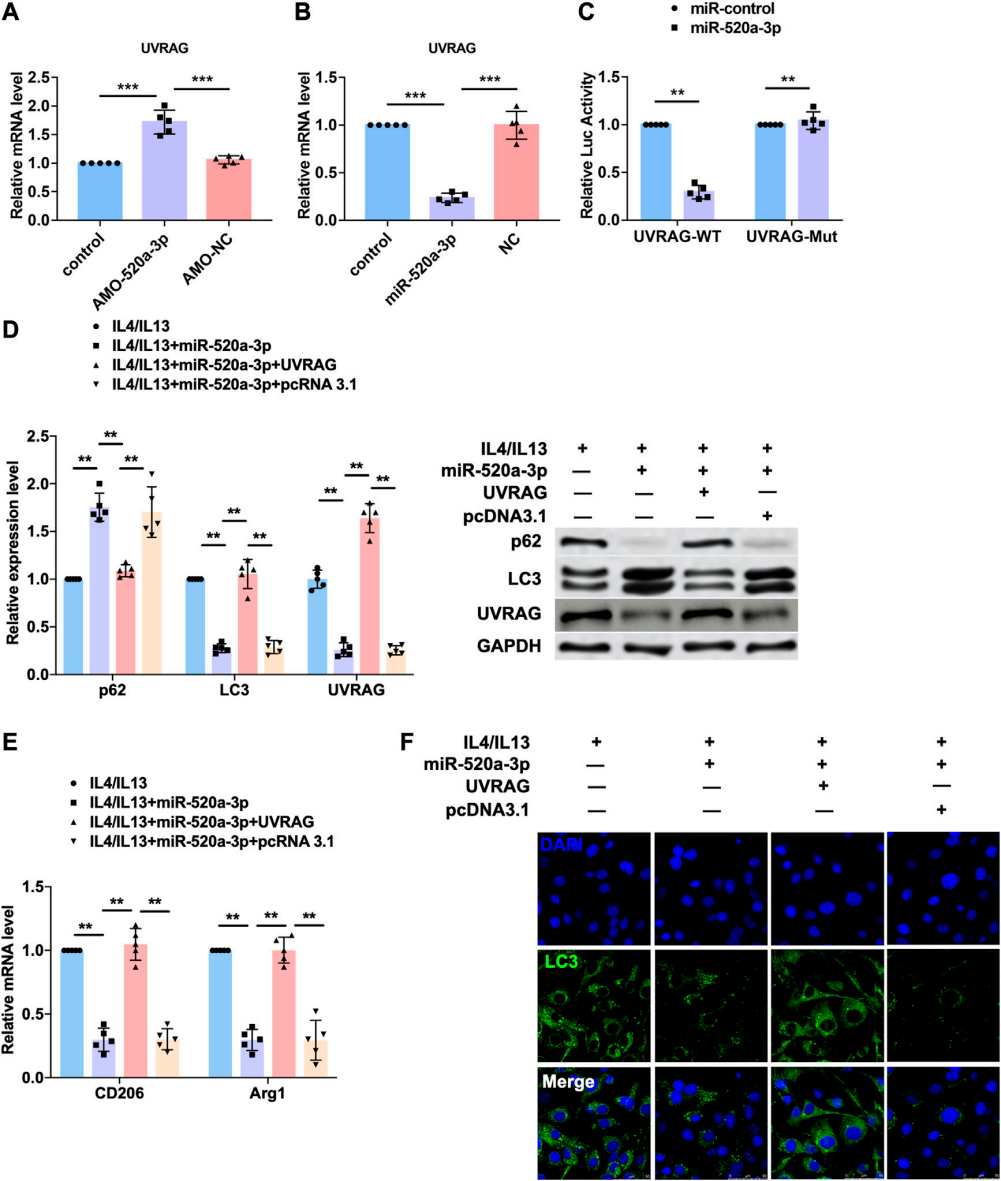
UVRAG mediated the inhibitory effect of miR-520a-3p on macrophage autophagy and polarization. **(A)** and; **(B)** RT-PCR analysis showed the mRNA level of UVRAG in macrophages. Data are mean ± SEM; one-way ANOVA was used for the statistical analysis. n=5 independent cell cultures; **(C)** Luciferase assay indicated the interaction of miR-520a-3p with 3’ UTR of UVRAG. Data are mean ± SEM; Two-tailed t test was used for the statistical analysis. n=5 independent cell cultures; **(D)** Western blot analysis showed the protein levels of p62, LC3 and UVRAG in macrophages. Data are mean ± SEM; one-way ANOVA was used for the statistical analysis. n=5 independent cell cultures; **(E)** RT-PCR analysis showed the mRNA level of CD206 and Arg1 in macrophages. Data are mean ± SEM; one-way ANOVA was used for the statistical analysis. n=5 independent cell cultures; **(F)** Immunofluorescence images showed the LC3 level in macrophages. Scale bars represent 50 μm. **P<0.01. ***P<0.001.

To predict the target gene of miR-520a-3p, we used targetscan (http://www.targetscan.org/vert_72/) and found UVRAG, a autophagy related gene that has been widely reported to mediated the activation of autophagy flux. So we performed western blot analysis to measure the protein level of UVRAG and found that inhibition of miR-520a-3p significantly elevated the expression of UVRAG in plaque and artery in apoE-/- mice (Supplementary Figure S1B). To explore whether UVRAG mediated the effect of miR-520a-3p in macrophage autopahgy, AMO-520a-3p and miR-520a-3p mimics were constructed to inhibit or overexpressed the expression of miR-520a-3p (Supplementary Figure S1A). As shown in Figure 4A B, silencing of miR-520a-3p elevated the mRNA level of UVRAG, which was inhibited by overexpression of miR-520a-2p. We then used luciferase assay to investigate the direct targeting between miR-520a-3p and UVRAG. 3’UTR of UVRAG was insert into the promoter sequence of luciferase gene. Treatment of miR-520a-3p significantly inhibited the luciferase activity, however, miR-520a-3p showed no effect on mutant 3’UTR of UVRAG group, which indicated the interaction between miR-520a-3p and UVRAG (Figure 4C). Next, we further determined whether miR-520a-3p play function via targeting UVRAG. IL4/IL13 induced inhibition of autophagy was inhibited by miR-520a-3p overexpression and forced expression of UVRAG abolished that effect (Figure 4D and Supplementary Figure S1C). In addition, overexpression of UVRAG significantly elevated the mRNA levels of CD206 and Arg1, which were reduced after miR-520a-3p transfection, and suggested that miR-520a-3p suppressed M2 macrophages polarization via inhibiting the expression of UVRAG (Figure 4E). immunofluorescence staining was also performed to examine the autophagy flux and UVRAG forced expression reversed the alleviation of autophagy induced by miR-520a-3p (Figure 4F).

## Discussion

Here we reported that miR-520a-3p was increased in mice with AS and silencing of miR-520a-3p attenuated AS process. Furthermore, inhibition of miR-520a-3p elevated the expression of collagen and α-SMA. In addition, miR-520a-3p silencing inhibited the expression of M1 macrophage polarization markers and pro-inflammatory genes and promoted the M2 macrophage polarization. What’s more, forced expression of miR-520a-3p diminished IL4/IL13 induced macrophage autophagy via targeting UVRAG.

Atherosclerosis, a kind of chronic inflammatory blood vessel disease, is a main cause of cardiovascular disease, which is a leading cause of mortality around the world (Frostegård, 2013). Initiation and development of atherosclerosis is dependent on local inflammation and accumulation of lipids in the vascular wall accompanied with the recruitment and infiltration of macrophages (Bäck et al., 2019). In the past ten years, the key role of noncoding RNA in physiological and pathological condition has been widely reported. Mountains evidence have revealed the role of miRNAs in regulation of AS progression (Feinberg and Moore, 2016). Previous study indicated that miR-520a-3p diminished gastric cancer cell glycolysis and proliferation by forming feedback with AKT1/mTOR/HIF1α pathway (Pan et al., 2019). What’s more, miR-520a-3p was also involved in the progression of different kinds of cancers (Li et al., 2017; Zhang et al., 2017; Lv et al., 2018; Bi et al., 2019). Here we reported that the expression level of miR-520a-3p was increased in AS mice. Inhibition of miR-520a-3p alleviated plaque areas and accumulation of lipids. In addition, silencing of miR-520a-3p increased the collagen deposition and the expression of α-SMA.

In the lesions of AS, macrophages are response to multiple kinds of stimuli, such as cytokines, inflammatory factors and so on (Jinnouchi et al., 2020). With the development of AS, pro-inflammatory cytokines trigger the recruitment of macrophages and promote the M1 type macrophage polarization of recruited macrophages and inhibit the M2 type macrophage polarization (Chinetti-Gbaguidi et al., 2015). Here we identified that silencing of miR-520a-3p inhibited the mRNA levels of pro-inflammatory genes containing TNF-α, IL6 and MCP-1 and reduced the expression of MI macrophage polarization related genes, IL1β and iNOS in mice with AS. In addition, inhibition of miR-520a-3p elevated the expression of CD206 and Arg1, meaning the promotion of M2 macrophage polarization.

Autophagy is a key intracellular process in maintaining cell homeostasis (Mizushima and Komatsu, 2011). Initiation of autophagy is induced by many stimuli, such as inflammation, hypoxia and accumulation of misfolding proteins (Ravanan et al., 2017; Racanelli et al., 2018). LC3I conjugated membrane is drove by ATG protein complex to elongate and form autophagosome with LC3I converted to LC3II (Li and Zhang, 2019). Misfolded proteins or impaired organelles are enveloped by autophagosome, which then fuses with lysosome and forms autolysosome to degrade these compositions and provide materials for cell biosynthesis (Wang and Le, 2019). Under pathological condition, cell autophagy also contributes to the change of gene expression profile. Activation of macrophage autophagy triggered by inflammation promotes the conversation of pro-inflammatory macrophage to M2 type anti-inflammatory macrophage polarization (Shao et al., 2016). We found that miR-520a-3p inhibited macrophage autophagy induced by treatment of IL4/IL13 and alleviated expression of M2 macrophage polarization related genes. The mechanism of miRNAs in different diseases has been well known. Here we predicted the target genes of miR-520a-3p and found UVRAG can be targeted by miR-520a-3p. gain- and loss-of-function experiments and luciferase assay indicated the targeting interaction between miR-520a-3p and UVRAG. Overexpression of UVRAG reversed the inhibitory effect of miR-520a-3p on macrophage autophagy and promoted M2 macrophage polarization. However, the upstream regulation of miR-520a-3p gene transcription is still need further investigated.

Taken together, we reported the promotive effect of miR-520a-3p on AS progression and provided a new target for the attenuation of AS lesion development and inflammation.

## Data Availability

The original contributions presented in the study are included in the article/Supplementary Material, further inquiries can be directed to the corresponding author.
