# Supplementary figures and images for: MiR-520a-3p Inhibited Macrophage Polarization and Promoted the Development of Atherosclerosis via Targeting UVRAG in Apolipoprotein E Knockout Mice
Source: Front Mol Biosci. 2021 Mar 9;7:621324. doi: 10.3389/fmolb.2020.621324 (PMC7985160; doi:10.3389/fmolb.2020.621324)

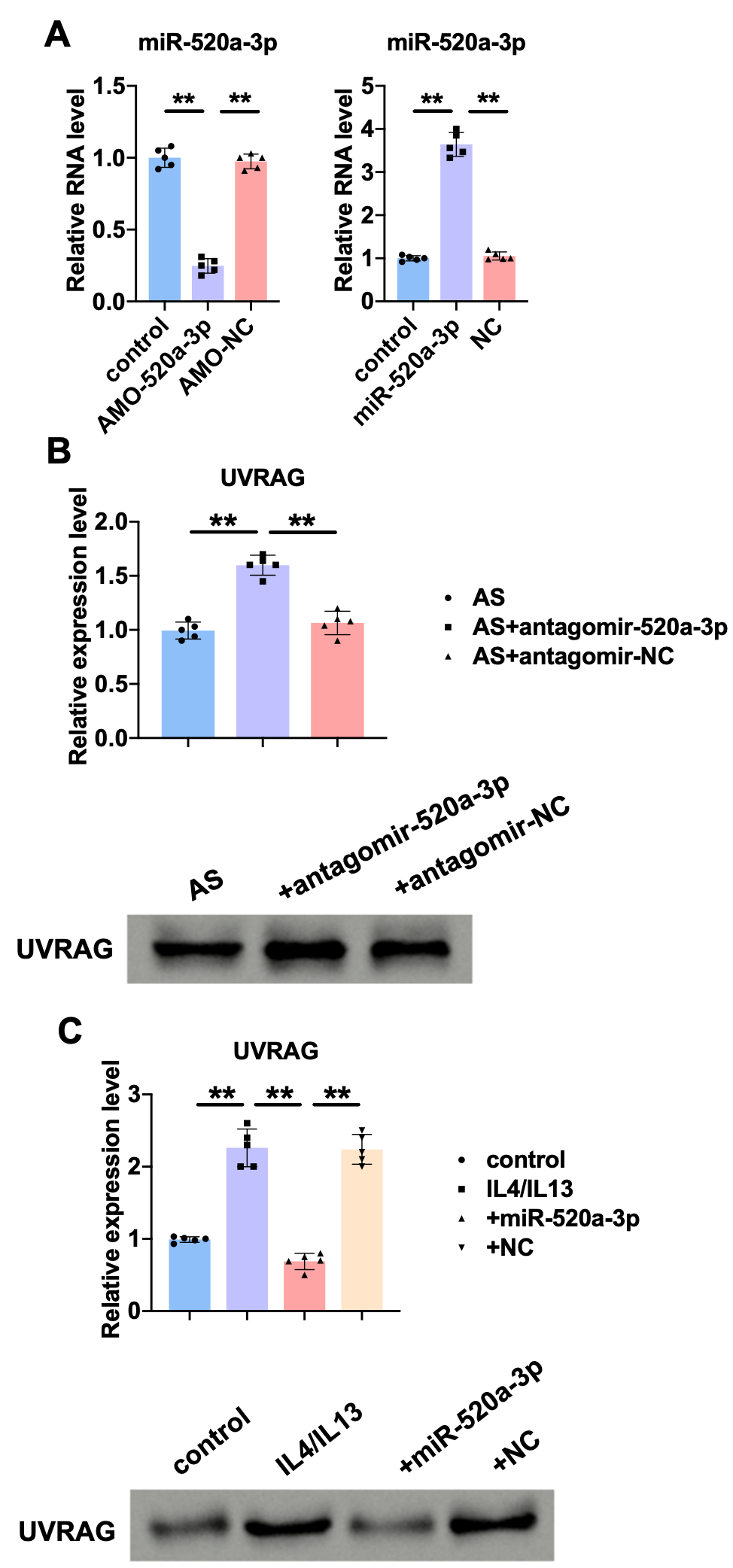

Supplement: Supplementary file 1 [file image1.tiff]
